# Indirect inguinal hernia containing omentum in an adult male: a clinical image

**DOI:** 10.11604/pamj.2026.53.16.49666

**Published:** 2026-01-15

**Authors:** Praveen Pawar, Archana Dhengere

**Affiliations:** 1 Department of Medical Surgical Nursing, Smt. Radhikabai Meghe Memorial College of Nursing, Datta Meghe Institute of Higher Education and Research, Wardha, Maharashtra, India

**Keywords:** Inguinal hernia, omentum, elective hernioplasty

## Image in medicine

A 45-year-old male presented with a reducible swelling in the right inguinal region that increased on coughing and standing. On examination, a positive cough impulse was noted, and the swelling extended towards the scrotum, suggestive of an inguinal hernia. The patient was taken for elective hernioplasty. Intraoperatively, the hernia sac was identified lateral to the inferior epigastric vessels, consistent with an indirect inguinal hernia. The sac contained omentum, which was reduced, and a Lichtenstein tension-free mesh repair was performed. The postoperative period was uneventful.

**Figure 1:**
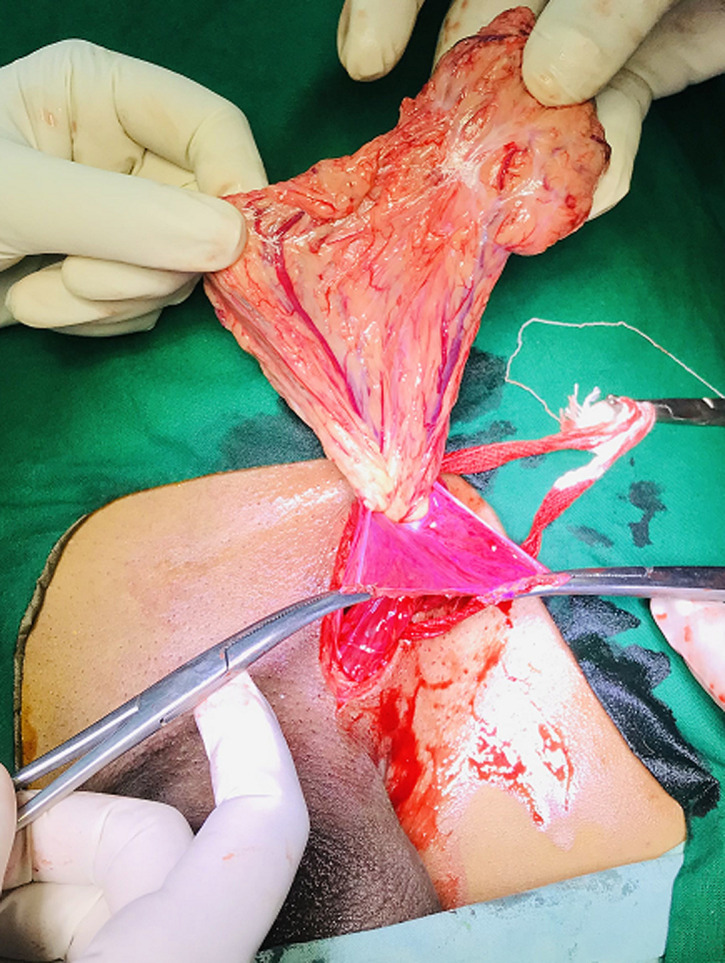
intraoperative image showing hernia sac containing omentum in an indirect inguinal hernia; note the sac being held up after dissection from surrounding structures

